# Medical Treatment for Endometriosis: Tolerability, Quality of Life and Adherence

**DOI:** 10.3389/fgwh.2021.729601

**Published:** 2021-09-27

**Authors:** Giussy Barbara, Laura Buggio, Federica Facchin, Paolo Vercellini

**Affiliations:** ^1^Obstetric and Gynaecologic Emergency Department and SVSeD (Service for Sexual and Domestic Violence), Fondazione IRCCS Ca' Granda Ospedale Maggiore Policlinico, Milan, Italy; ^2^Department of Clinical Sciences and Community Health, University of Milan, Milan, Italy; ^3^Gynaecology Unit, Fondazione IRCCS Ca' Granda Ospedale Maggiore Policlinico, Milan, Italy; ^4^Department of Psychology, Catholic University of the Sacred Heart, Milan, Italy

**Keywords:** endometriosis, medical treatment, estrogen-progestins, GnRH agonists, GnRH antagonists, quality of life, tolerability

## Abstract

Endometriosis is associated with painful symptoms, infertility, sexological difficulties, and psychological suffering. All these symptoms have a negative impact on the overall quality of life of women with the disease, with significant personal, social and economic costs. Several medical options are available to manage symptomatic endometriosis. The pharmacological treatment for endometriosis-related pain may be necessary for decades, or at least until there is a desire for pregnancy or physiologic menopause occurs. In this perspective, clinicians should consider not only the efficacy, but also side effects, tolerability, and costs, along with women's preferences toward different treatments. In this mini-review, we analyzed the pros and cons of the available drugs for the medical therapy of endometriosis, such as estrogen-progestins, progestins, GnRH agonist and GnRH antagonists.

## Introduction

Several medical options are available to manage symptomatic endometriosis, a chronic inflammatory estrogen-dependent disease characterized by the presence and proliferation of endometrium outside the uterine cavity (1–4). Endometriosis is associated with painful symptoms such as chronic pelvic pain, dysmenorrhea and dyspareunia, infertility, sexological difficulties, and psychological suffering (5–11). All these symptoms have a negative impact on the overall quality of life of women with endometriosis, including their social (12) and sexual relationships (9, 10, 13), work and study productivity (14–16), with remarkable social and economic costs (17, 18).

The rationale underpinning medical treatment for endometriosis is that endometriotic foci respond to ovarian steroids similarly to the eutopic endometrium (19), despite the presence of endometriosis-associated peculiar endocrine pathways, such as for instance progesterone resistance (20, 21). Thus, from a general perspective, the different pharmacological compounds available for treating endometriosis act by interfering with the pituitary-gonadal axis, determining a hypoestrogenic state, inducing anovulation, and reducing or suppressing the amount of menstrual flow (19).

By definition, medical therapy for endometriosis is symptomatic and not curative, as the pharmacological approach is not cytoreductive and the hypo-estrogenic milieu determined by the hormonal suppression is temporary (1, 3, 19). Consequently, the effect of the drug is expected to end after treatment discontinuation. However, this does not mean that medical therapy is ineffective. Like other chronic illnesses, such as hypertension or diabetes, the efficacy of pharmacological therapy on endometriosis-related symptoms should be assessed during treatment and not after discontinuation (1, 19).

Pelvic pain, in particular dysmenorrhea and dyspareunia, significantly affects women's experience of endometriosis and their quality of life (8). It has been demonstrated that women suffering from endometriosis-associated pelvic pain had poorer quality of life and psychological health as compared with women with asymptomatic endometriosis and the healthy controls (8).

The medical treatment of endometriosis can ameliorate painful symptoms of the disease and, consequently, reduce the negative impact on quality of life and on mental health.

In addition, it should be considered that the pharmacological treatment for endometriosis-related pain may be necessary for years, or at least until there is a desire for pregnancy or physiologic menopause occurs.

This also means that drugs for endometriosis-related pain should have a high safety profile, be well-tolerated, have few side effects, and have reasonable costs (22). Moreover, pharmacological therapies for endometriosis prevent pregnancies during their use and do not increase the likelihood of conception after their discontinuation. Therefore, women should be informed that medical therapies for endometriosis have no role in case of infertility (1). Thus, medical therapy for endometriosis should be proposed to women with endometriosis-related pain with no wish for pregnancy and without surgical indications. Absolute indications for surgery include the presence of large endometriomas, adnexal masses of uncertain appearance at diagnostic imaging procedures, ureteral stenosis causing hydronephrosis, and bowel stenosis associated with sub-occlusive symptoms (19).

According to several guidelines on endometriosis-management released by the most authoritative gynecological societies (23–26), hormonal contraceptives, progestins, anti-progestogens, GnRH agonists, and GnRH antagonists should be used for the management of endometriosis-related pain.

A pragmatic and reasonable approach to medical treatment for endometriosis-related pain should involve a realistic balance with a long-term view between efficacy (in terms of improvement of the overall quality of life of women affected by the disease), safety, tolerability, and costs, considering women's treatment preferences and their wish for pregnancy.

### Estrogen-Progestins and Progestins

Combined hormonal contraceptives have been used for many years as first-line therapy for symptomatic endometriosis. Estradiol has shown antiapoptotic and inflammatory effects on ectopic endometrial tissue, whereas progestins have anti-inflammatory and pro-apoptotic properties (27).

The combined oral contraceptives currently in use contain a low level of ethinylestradiol, and have a prevalent progestin effect on ectopic endometrial tissue. In addition, they reduce or completely abolish the menstrual flow, thus limiting the amount of trans-tubaric reflux of endometrial cells. This should result in a reduction of the associated pelvic oxidative stress and inflammation induced by free peritoneal iron and heme resulting by erythrophagocytosis and lysis of erythrocytes by pelvic macrophages (28). Thus, estrogen-progestins induce atrophy of the eutopic and ectopic endometrium, limit retrograde menstruation, inhibit ovulation, and have anti-inflammatory and proapoptotic effects on endometriotic foci. Several studies (1, 19, 29–31) have shown that at least two-thirds of women affected by endometriosis-related pelvic pain will benefit from the use of estrogen-progestins combinations, in particular with regards to dysmenorrhea, with a reported significant amelioration in the overall quality of life (29–31). However, one-third of the women with endometriosis do not respond to estrogen-progestins, which may be in part due to progesterone resistance (32).

Estrogen-progestins can be delivered through different modalities (1, 33) – such as oral, vaginal, or transdermal – according to women's preferences. The different methods of hormonal administration, which allow for a daily or weekly drug administration, can increase patients' compliance with treatment and adherence (31), which is particularly important when a pharmacological treatment is required for a long time, as is the case of a chronic disease such as endometriosis (19). In addition, estrogen-progestins represent the only treatment for endometriosis that allows the occurrence of cyclic uterine bleeding. Monthly bleeding seems important for those women who strongly believe that amenorrhea represents a non-physiological state. Also this characteristic can increase women's compliance with treatment and adherence.

If dysmenorrhea persists with a cyclic use, women should be invited to use estrogen-progestins continuously, and to tailor the cyclic suspension of the hormonal treatment based on the occurrence of spotting/breakthrough bleeding, as well as their preferences and needs (30). An estrogen-progestin combination containing the lowest dose of estrogen and a second-generation progestin should be preferred to combine an optimal endometriosis suppression with minimization of the thrombotic risk (1, 19). Moreover, the most frequent adverse effects of estrogen-progestins should be explained to women, along with the other potential therapeutic alternatives (i.e., other pharmacological treatments, such as progestins, GnRH agonists and antagonists, or surgery), in order to increase women's awareness of therapeutic choices and adherence to treatment.

The European Society of Human Reproduction and Embryology (ESHRE) guidelines on endometriosis (24) pointed out that, although the evidence on the use of estrogen-progestins for endometriosis is limited, combined hormonal contraception is extensively used as a treatment for endometriosis-associated pain. This may be due to pragmatic and reasonable benefits of the estrogen-progestins therapy in women affected by endometriosis, including contraceptive action, few side effects, long term safety and good control of uterine bleeding. All these characteristics of the estrogen-progestins have a positive impact on the overall tolerability of the medical treatment and in general on patients' quality of life.

Progestins (depot medroxyprogesterone acetate, medroxyprogesterone acetate, norethisterone acetate, desogestrel and dienogest) can be used as second line treatments (1). These compounds could represent a reasonable option in women with endometriosis who do not respond to estrogen-progestins (1, 19) or in case of deep endometriotic lesions or in the presence of deep dyspareunia (29, 33, 34). Moreover, progestins can be safely used in women with contraindications to the assumption of estrogens (35), as well as in those who do not tolerate estrogens because of their side effects (36).

Progestins can be delivered through different modalities (i.e., via oral, intramuscular, subcutaneous, or intrauterine route) to increase women's' compliance with treatment and adherence. All available progestins seem to be similarly effective in managing endometriosis-related pelvic pain and ameliorate painful symptoms and the overall quality of life in about two-thirds of women affected by the disease (33). As there is no clear evidence indicating the superiority of one progestin over the others, some authors (19, 33) suggested using norethisterone acetate (NETA) as the first therapeutic choice because of its safety and favorable cost-effectiveness profile. In particular, oral NETA (at the dose of 2.5 and 5 mg per day) (29, 37–43) was demonstrated to be effective in patients with deep endometriosis, such as rectovaginal or colorectal lesions (29, 38, 39, 43). In these women, the reduction in the degree of deep dyspareunia during the treatment with oral NETA was gradual but progressive over time (40).

As NETA is partially metabolized to estradiol (44), unfavorable effects on bone mineral density due to a prolonged treatment were not observed. The most frequent side effects associated with the use of NETA are weight gain, acne and seborrhea, related to the androgenic activity of this compound or to the occurrence of erratic bleeding. In case of frequent or persistent breakthrough bleeding, amenorrhea can be achieved suggesting women discontinuing treatment for some days (30, 31). Providing such information to women appears essential in order to improve adherence and satisfaction with therapy.

Dienogest, 2 mg/day per os, constitutes a valid alternative in those women who experience androgenic side-effects during NETA use. In fact, due to its mild anti-androgenic properties, dienogest appears better tolerated then NETA (42), and its use may increase tolerability and treatment adherence in a large number of patients.

In general, although side effects associated with progestins are relatively frequent, about 70% of women are satisfied with this therapy. Thus, high adherence and low dropout rates have been reported with the use of progestins for the management of endometriosis-associated pain (1, 19).

### GnRH Agonists and Antagonists

GnRH agonists are very effective for treating endometriosis-associated pain (24), despite their limited tolerability and safety. GnRH agonists create a deep reversible hypoestrogenic state, and evidence regarding dosage and duration of treatment is limited (24). Side effects, such as hot flushes, sleep disturbance, and mood swings, are persistent and caused by the severe hypoestrogenic state induced by these drugs. Adding an add-back therapy (i.e., low-dose progestins or tibolone) should be strongly suggested to minimize the frequency of climacteric-like symptoms and improve tolerability and adherence to therapy (24). In fact, the use of GnRH agonists as a monotherapy, especially in young women and adolescents, is limited by the unfavorable long-term safety profile, as well as by the frequency and severity of side effects.

An oral GnRH antagonist (elagolix) was recently marketed for treating women with endometriosis (45, 46). The mechanism of action of elagolix is a dose-dependent suppression of the ovarian estradiol production, and therefore the induction of a certain degree of hypoestrogenic state, avoiding the flare-up phase, typically associated with the use of GnRH agonists. Elagolix, at the oral daily dose of 150 or 400 mg, was found to determine a reduction in dysmenorrhea of about 46% in the lower-dose group and 76% in the higher-dose group, as compared to a menstrual pain reduction of about 23% in the placebo group (46). At 6-month follow-up, 47–66% of women taking the higher elagolix dose of 400 mg experienced amenorrhea. Hot flushes were reported by 42–48% of women in the 400 mg elagolix-dose group. As elagolix does not completely suppress ovulation, women should be instructed to use non-hormonal contraception. However, unplanned pregnancies were observed in women who used elagolix (46). In theory, the need for barrier contraception and the fear of unplanned pregnancies may limit women's adherence to treatment.

Other GnRH antagonists are currently being evaluated for the treatment of endometriosis, such as relugolix and linzagolix (47–50). In view of the above considerations, all these oral GnRH antagonists are now being studied in association with low-dose estrogen-progestin combinations, similarly to the add-back therapies used with GnRH agonists.

It would be very helpful for women with endometriosis if GnRH antagonists will be compared with progestins in pragmatic trials. This would allow the definition of the incremental benefit of these novel drugs compared with first-line medications not only in terms of efficacy on pain symptoms and amelioration of the women's quality of life, but also of tolerability and adherence. Moreover, the cost of any long-term medical treatment has been demonstrated to be a determinant factor for adherence (51).

## Conclusions

In conclusion, when choosing medical treatments for endometriosis-related pain, clinicians should consider not only the efficacy, but also side effects, tolerability, adherence to treatment, costs and women's preferences (see also Table 1 for more details on tolerability, adherence and quality of life in the context of endometriosis). This appears. This appears particularly important if one considers the chronic nature of the disease, potentially determining a long-term impairment of women's overall quality of life, mental health, social activities, work, sexual and intimate relationships. Oral very-low-dose monophasic estrogen-progestin combinations may be considered for women with peritoneal lesions and endometriomas, whereas progestins should be favored for those with deep infiltrating lesions (1).

**Table 1:** Tolerability, quality of life, and adherence in the context of endometriosis.

Tolerability	Tolerability to a certain pharmacological therapy refers to the extent to which adverse effects can be tolerated by a patient. This could be particularly important in the management of chronic diseases, such as endometriosis, for which treatment may be necessary for years. Typically, tolerability can be defined by the rate of “dropouts”, or women affected by endometriosis that forfeit participation in a clinical study due to extreme adverse effects.
Quality of life	Quality of life is defined by the World Health Organization as “individuals' perception of their position in life in the context of the culture and value systems in which they live, and in relation to their goals, expectations, standards and concerns”. (https://www.who.int/tools/whoqol). Quality of life has become a major and crucial aim of current health-care, in particular as regard to painful chronic diseases, such as endometriosis.
Adherence	Adherence to a certain pharmacological therapy refers to the extent to which the patient's behavior fulfills with the recommendations provided by the health care professionals. Several factors can affect adherence to a certain treatment, including for instance poor provider-patient communication, fear of severe adverse effects, complex therapies that require various medications, long-term drug regimens, economic barriers. In the context of endometriosis, to improve woman medication adherence, it should be important that physicians recognize specific concerns for each woman in relation to a certain therapy, and implement appropriate personalized procedures to overcome them.

The heterogeneity of the studies in relation to quality of life, adherence and tolerability (in terms of research methods, types of endometriosis, and types of questionnaires used to assess quality of life outcomes) make it difficult to obtain firm conclusions. We strongly believe that researchers, in designing clinical trials in the context of endometriosis, should consider as main outcomes aspects related to women's quality of life, measured by validated standardized instruments to facilitate comparisons. Moreover, future studies in the pharmacological treatment of endometriosis should focus on comparison trials with other progestins or estrogen-progestins, and should be designed as superiority trials (33).

A tailored shared-decision making process should guide the clinicians in the selection of the appropriate treatment. The choice between different available therapeutic options should be pondered considering lesion type and location, the most severe reported symptom, and the need for contraception (33).

The rationale that should guide the clinicians in the management of women with endometriosis, as stated by the Practice Committee of the American Society of Reproductive Medicine (ASRM) (23), is the maximization of the use of medical therapies for long periods of time in order to achieve adequate control of pain symptoms and amelioration of quality of life, and to minimize the use of repeated surgery.

## Author Contributions

PV and GB conception and design of the article. GB drafting the article. GB, LB, FF, and PV literature review and acquisition of data. PV and LB revising the article for intellectual content. All authors contributed to the article and approved the submitted version.

## Funding

This work was supported by “Ricerca Corrente 2021”, Italian Ministry of Health.

## Conflict of Interest

The authors declare that the research was conducted in the absence of any commercial or financial relationships that could be construed as a potential conflict of interest.

## Publisher's Note

All claims expressed in this article are solely those of the authors and do not necessarily represent those of their affiliated organizations, or those of the publisher, the editors and the reviewers. Any product that may be evaluated in this article, or claim that may be made by its manufacturer, is not guaranteed or endorsed by the publisher.
